# Apparent and standardized ileal nutrient digestibility of broiler diets containing varying levels of raw full-fat soybean and microbial protease

**DOI:** 10.1186/s40781-017-0148-2

**Published:** 2017-10-16

**Authors:** Mammo M. Erdaw, Rider A. Perez-Maldonado, Paul A. Iji

**Affiliations:** 1 School of Environmental and Rural Sciences, University of New England, Armidale, NSW 2351 Australia; 2 DSM Nutritional Products, Animal Nutrition and Health, 30 Pasir Panjang Road #13-31 Mapletree, Business City, 117440 Singapore; 3 Ethiopian Institute of Agricultural Research, Addis Ababa, Ethiopia

**Keywords:** Amino acids, Antinutritional factors, Broilers, Ileal digestibility, Microbial protease, Trypsin inhibitors

## Abstract

**Background:**

Although soybean meal (SBM) is excellent source of protein in diets for poultry, it is sometimes inaccessible, costly and fluctuates in supply. The SBM can partially be replaced by full-fat SBM, but the meals prepared from raw full-fat soybean contain antinutritional factors. To avoid the risk of antinutritional factors, heat treatment is always advisable, but either excessive or under heating the soybean could negatively affect the quality. However, the potential for further improvement of SBM by supplementing with microbial enzymes has been suggested by many researchers. The objective of this study was to evaluate the performance and ileal nutrient digestibility of birds fed on diets containing raw soybeans and supplemented with microbial protease.

**Methods:**

A 3 × 2 factorial, involving 3 levels of raw full-fat soybean (RFFS; 0, 45 or 75 g/kg of diet) and 2 levels of protease (0 or 15,000 PROT/kg) was used. The birds were raised in a climate-controlled room. A nitrogen-free diet was also offered to a reference group from day 19 to 24 to determine protein and amino acid flow at the terminal ileum and calculate the standardized ileal digestibility of nutrients. On days 10, 24 and 35, body weight and feed leftover were recorded to calculate the body weight gain (BWG), feed intake (FI) and feed conversion ratio (FCR). On day 24, samples of ileal digesta were collected at least from two birds per replicate.

**Results:**

When RFFS was increased from 0 to 75 g/kg of diet, the content of trypsin inhibitors was increased from 1747 to 10,193 trypsin inhibitors unit (TIU)/g of diets, and feed consumption of birds was also reduced (*P* < 0.05). Increasing RFFS level reduced the BWG from hatch 0 to 10 d (*P* < 0.01) and hatch to 24 d (*P* < 0.05). The BWG of birds from hatch to 35 was not significantly (*P* = 0.07) affected.

Feed intake was also reduced (*P* < 0.05) during 0 to 35 d. However, protease supplementation improved (*P* < 0.05) the BWG and FCR during 0 to 24 d. Rising levels of RFFS increased the weight of pancreas (*P* < 0.001) and small intestine (*P* < 0.001) at day 24. Except for methionine, apparent and the corresponding standardized ileal digestibility of CP and AA were reduced (*P* < 0.01) by increasing levels of RFFS in diets.

**Conclusion:**

This study showed that some commercial SBM could be replaced by RFFS in broiler diets, without markedly compromising productivity. The AID and SID of CP and lysine were slightly improved by dietary supplementation of microbial protease.

## Background

It is well established that commercial soybean meal (SBM) is an excellent source of protein in diets for poultry [1]. However, in addition to the fluctuation in supply and seasonal scarcity in some parts of the world, the price of SBM has been increasing over the years [1, 2]. Poultry producers therefore continuously seek alternative ingredients, including full-fat SBM to replace some or all the commercial SBM in diets for broilers [3]. Full-fat soybean meal is typically made from heat-treated seeds; and it is less common to feed soybean as raw. Such processing plants are lacking in some areas of the world where soybeans are locally produced and poultry producers could save some costs if the raw soybean could be fed. However, raw soybean seeds contain considerable amounts of ANF, particularly trypsin inhibitors (TI), which depress growth in non-ruminant animals [4–7]. The presence of dietary TI in legumes, such as soybeans causes a substantial reduction in the digestibility (up to 50%) of proteins and AA and protein quality (up to 100%) in non-ruminant animals [8]. Nitrogen (N) retention can also be negatively affected by TI causing an increased endogenous N loss [9, 10]. Although Barth et al. [11] reported that inclusion of RFFS in diets caused loss of endogenous protein, Clarke and Wiseman [12] reported that AA digestibility did not correlate with levels of RFFS supplementation/concentration of TI.

Although heating is considered to be the most effective method to eliminate or reduce ANF, some of the ANF in soybeans, such as the Bowman-Birk inhibitors, phytates and oligosaccharides are not heat-labile. These ANF remain a problem in feeds prepared from raw soybean grains and poorly processed commercial meals. Clemente et al. [13] also reported that Bowman-Birk inhibitors exhibit resistance to heat treatment, so that supplementation of such corn-soybean meal diets with microbial enzymes including protease and phytase is necessary [10, 14].

The potential for further improvement in the nutritional value of soybeans using exogenous enzymes has therefore been suggested by many researchers [5, 12, 15, 16]. The negative effects of microbial protease supplementation of poultry diets have been widely documented [17–19]. However, the role of such enzymes on RFFS has not been adequately investigated. In recent in vitro and in vivo studies, we reported the positive response of protein and phytate in RFFS to microbial protease and phytase and gross response of poultry on such test diets [20–23]. The objective of the current study was to assess how these enzymes, particularly protease, affect CP and AA digestibility.

## Methods

### Diets, experimental design and animal husbandry

The experiment was approved by the University’s Animal Ethics Committee (Authority No: AEC15–044) and conducted at the Animal House of the University of New England, Australia. The soybean grain was purchased from a local supplier in northern New South Wales, Australia. After cleaning and hammer-milling the grain to pass through a 2-mm sieve size, the meal was used to partially replace the commercial SBM at 0, 15 or 25%, equivalent to 0, 45 and 75 g/kg diet, respectively (Table 1). Birds were offered corn-soybean meal-based starter (0 to 10 d), grower (10 to 24 d) and finisher (24 to 35 d) diets, which were formulated to the breeder standard for Ross 308 broilers [24]. The nutrient requirement of birds across the dietary treatment was balanced by supplementing with varying levels of canola oil, synthetic AA and the meat meal. The diets were supplemented with phytase (HiPhos) at 2000 phytase activity (FYT)/kg, equivalent to 0.2 g/kg of diet, and fed as such or further supplemented with protease (ProAct) at 15000 PROT/kg (15,000 units of protease/kg diet), the level approved by the European Food Safety Authority [25]. These enzymes were supplied by DSM Animal Nutrition, Asia-Pacific, Singapore. The microbial protease was added prior to pelleting the diets. Titanium dioxide was added to the grower diets to enable assessment of nutrient digestibility. Feed, in the form of crumble (starter) and pellet (grower and finisher periods) was provided ad libitum, and the birds had free access to water. Samples of the diets (with or without protease) were analyzed to evaluate the contents of CP and AA (Table 2).

**Table 1:** Ingredient and composition of starter, grower and finisher basal, and a nitrogen-free diet

Item	Starter			Grower			Finisher			Nitrogen-free diet
	RFFS^1^, g/kg			RFFS^1^, g/kg			RFFS^1^, g/kg			
	0	45	75	0	45	75	0	45	75	
Ingredients (g/kg)										
Corn (rolled)	594.0	595.0	592.0	579.0	581.0	576.5	607.0	608.2	610.0	0.0
Corn starch	0	0	0	0	0	0	0	0	0	433.0
Dextrose	0	0	0	0	0	0	0	0	0	404.0
Soybean meal	300	255	225	300	255	225	300.0	255.0	225.0	0.0
Raw soybean	0	45	75	0	45	75	0	45.0	75.0	0
Canola oil	16.0	9.4	7.9	45.6	40.5	40.3	51.1	46.3	43.5	50.0
Meat meal	62.9	67.1	72.3	39.0	42.1	46.0	6.0	9.0	10.0	0.0
Cellulose	0.0	0.0	0.0	0.0	0.0	0.0	0	0	0	50.0
Dicalcium phosphate	7.7	8.0	7.6	9.5	11.0	11.0	15.8	15.5	15.5	25.7
TiO_2_	0	0	0	5.0	5.0	5.0	0	0	0	5.0
Limestone	6.2	6.5	6.3	10.0	7.5	8.5	11.0	9.8	10.0	11.9
Salt	3.0	3.0	3.0	2.3	2.0	2.3	2.2	2.1	2.1	0.0
L-lysine	2.7	2.9	2.7	2.0	2.4	2.2	0.7	1.0	1.0	0.0
DL-methionine	2.3	2.1	2.5	3.3	3.8	3.7	2.2	2.7	2.8	0.0
Premix^2^ (g/kg)	2.0	2.0	2.0	2.0	2.0	2.0	2.0	2.0	2.0	2.0
L-threonine	2.0	2.0	2.0	0.6	1.5	0.9	0.5	1.8	1.8	0.0
Sodium chloride	0	0	0	0	0	0	0	0	0	2.5
Sodium bicarbonate	1.4	1.6	1.4	1.1	1.0	0.9	1.0	1.0	1.0	1.9
Magnesium sulphate	0	0	0	0	0	0	0	0	0	1.2
Potassium chloride	0	0	0	0	0	0	0	0	0	9.0
Choline chloride	0.5	0.5	0.5	1.0	0.5	0.8	0.5	0.5	0.5	3.5
Phytase (g/kg)	0.20	0.20	0.20	0.20	0.20	0.20	0.20	0.20	0.20	0
Nutrients (g/kg)										
Metabolizable energy (poultry; MJ/kg)	12.59	12.59	12.59	13.28	13.28	13.28	13.49	13.49	13.49	14.44
Crude protein	225.8	225.8	226.0	210.0	210.7	208.6	192.1	192.9	190.5	<6.0
Crude fat	42.1	39.8	44.6	68.7	67.7	73.6	68.8	70.7	71.9	0
Arginine	14.4	14.3	14.4	13.4	13.3	13.3	12.3	12.3	12.1	0
Lysine	14.0	14.0	14.0	12.7	12.7	12.8	10.5	10.7	10.6	0
Methionine	5.7	5.2	5.7	6.5	6.7	6.6	5.0	5.4	5.4	0
Met + Cys	8.8	8.3	8.8	9.4	9.7	9.6	7.9	8.3	8.3	0
Threonine	9.9	9.4	9.9	8.2	8.9	8.3	7.4	8.7	8.6	0
Calcium	10.0	10.3	10.0	9.9	9.4	9.8	8.7	8.5	8.5	10.0
Available phosphorus	5.0	5.1	5.0	4.4	4.6	4.6	4.2	4.3	4.2	4.8
Choline	1.4	1.4	1.4	1.5	1.3	1.4	1.4	1.3	1.2	1.5

^2^Premix (g/kg of feed): Cu, 8 mg; Fe, 60 mg; I, 1.0 mg; Se, 0.3 mg; Mn, 80 m g; Zn, 60 mg; Mo, 1 mg; Co, 0.3 vitamin A, 12 MIU; Vitamin D_3_, 3.5 MIU; Vitamin E, 40 g, Vitamin K_3_, 2 mg; thiamine, 2 mg; riboflavin (B_2_), 6 mg; niacin (B_2_), 50 mg; pantothenate, 11 mg; pyridoxine, 20 mg; folate, 0.0015 mg; Biotin, 100 mg; Vitamin B_12_, 0.02 mg; Vitamin B_6_; 1.5 mg; biotin, 0.01 mg; Antioxidant, 25 mg. ^1^RFFS = raw full-fat soybean. SBM = commercial soybean meal. Basal diets were prepared when the SBM was replaced by RFFS at 0, 15 and 25%, equivalent to 0, 45 and 75 g/kg of diet, respectively)

Microbial protease was then supplemented to the basal diets (0 or 15,000 PROT/kg of diet). A 15000 PROT/kg = 15,000 units of protease/kg of diet, equivalent to 200 mg of protease/kg of diet. The 3 experimental periods of this study were starter (day 0 to 10), grower (day 10 to 24) and finisher (day 24 to 32). FYT = FTU as a unit that measuring the releasing capacity of nutrient by microbial phytase. NFD = nitrogen free diet

**Table 2:** The analysed crude protein (CP) and amino acid composition (g/kg) of the study diets fed in days of 10 to 24

Protease (PROT/kg)	0			15,000			Nitrogen-free diet
RFFS^1^ (g/kg)	0	45	75	0	45	75	0
Crude protein	224	219	222	224	221	217	<6.0
Indispensable amino acids							
His	5.5	5.3	5.5	5.5	5.4	5.2	<0.1
Arg	14.3	14.2	14.4	14.1	14.4	13.0	<0.4
Thr	8.7	8.4	8.8	9.2	9.2	8.4	<0.2
Lys	12.8	12.3	13.4	12.8	13.3	11.9	<0.3
Met	5.1	4.7	5.2	5.8	5.3	5.0	<0.1
Val	10.7	10.2	10.7	10.5	10.4	10.0	<0.3
Ile	9.0	8.7	9.1	8.8	8.8	8.2	<0.2
Leu	18.0	17.6	18.1	17.9	18.0	17.9	<0.5
Phe	10.4	10.1	10.4	10.3	10.3	9.8	<0.3
Dispensable amino acids							
Ser	10.6	10.4	10.6	10.5	10.5	10.1	<0.3
Gly	11.4	12.4	11.4	10.6	11.1	10.4	<0.3
Asp	20.3	20.1	20.7	19.8	20.4	19.0	<0.5
Glu	37.2	37.0	37.8	36.8	37.6	36.2	<1.0
Ala	11.0	11.2	11.0	10.7	10.9	10.8	<0.3
Pro	13.6	14.0	13.6	13.3	13.5	13.2	<0.4
Tyr	4.9	5.0	5.2	5.0	5.1	4.4	<0.1

^1^RFFS = raw full-fat soybean (SBM was replaced by RFFS at zero, 15 and 25%, equivalent to zero, 45 and 75 g/kg of diet, respectively). A 15000 PROT/kg = 15,000 units of protease/kg of diet, equivalent to 200 mg of protease / kg of diet

A total of 336 Ross 308 male broiler chicks (43.84 ± 0.18 g) were obtained from a local commercial hatchery (Baiada Poultry Pty. Ltd., Tamworth, Australia). These birds were randomly selected, weighed and randomly allocated into 42 pens at eight chicks per pen. This was a 2 × 3 factorial study, with each treatment being replicated six times and eight birds per replicate. Another lot of 48 birds, in six replicates, were fed on a nitrogen-free diet (NFD).

The six replicates were provided with the commercial-types of starter and grower diets before they were transferred to the NFD diet, which was prepared without RFFS or microbial enzymes. The birds were raised in a climate-controlled room on sawdust litter.

On day 19, a total of 48, with 8 birds per replicate were transferred to a nitrogen-free diet (NFD) to enable calculation of CP and AA flow at the ileum, and estimate standardized ileal digestibility (SID) of these nutrients.

Every pen was equipped with a clean feeder and two nipple drinkers that were daily checked and cleaned. The room temperature was set at 33 °C for the first two days, with a relative humidity of between 49 and 60%. The temperature was gradually reduced to 24 °C at 19 days of age and was maintained for the remaining study period. Lighting was provided for 24 h (20 lx) for the first two days, then reduced to 23 h for the next 6 consecutive days, followed by 20-h light (10 lx) for the remaining days. Mortality of birds was recorded whenever it occurred.

### Data collection

On days 10, 24 and 35, the body weight of birds and feed leftover were recorded, to calculate the body weight gain (BWG) and feed intake (FI), from which the feed conversion ratio (FCR) was computed. On day 24, at least two birds per replicate were euthanised by cervical dislocation and excised. Ileal digesta were collected on ice, pooled per replicate, and then transferred to a freezer (−20 °C) until they were analyzed for nutrient composition. Except for birds on NFD, samples of internal organs were also collected at day 24 and weighed. One representative bird per cage was randomly selected, electrically stunned and killed by cervical dislocation. The bird was dissected to obtain internal organs, which were weighed as described by [26]. The remaining birds, (except those allocated to the NFD) were transferred to finisher diets and raised to 35 day of age, with the aim of evaluating the growth performance.

### Chemical analysis and calculation of nutrient digestibility

Sub-samples of the ingredients and test diets were analysed for CP and AA [27, 28], urease activity (UA) [29], nitrogen solubility index (NSI) [30], contents of TI (TIU/g) [31], protein solubility [32], starch [33], total sugars [33], ether extract [34] and crude fiber [35].

According to Cohen and Michaud [36, 37], amino acid contents of ingredients, diets and digesta were analyzed by the Australian Proteome Analysis Facility, Macquarie University, Australia. Amino acid (AA) concentrations were determined using pre-column derivatization AA analysis with 6-aminoquinolyl-*N*-hydroxysuccinimidyl carbamate followed by separation of the derivatives and quantification by reversed phase high performance liquid chromatography.

The concentration of titanium (Ti) in the ileal digesta and diets was determined using the method described by Short et al. [38]. The data on concentrations of nutrients and the Ti marker were used in the following calculations.

Ileal AA outflow (IAAF; mg/g intake) and ileal CP outflow (ICPF; mg/g intake) for all treatments (including NFD) were determined against the Ti concentration as follows:

IAAF or ICPF = AA or CP in digesta (mg/g)/[Ti in diet (mg/g)/Ti in digesta (mg/g)].

The coefficient of apparent ileal digestibility (AID) and the coefficient of standardized ileal digestibility (SID) of CP and AA were calculated using the following equations:

$$ \mathrm{AID}=\left(\mathrm{diet}\ \mathrm{AA}\ \mathrm{or}\ \mathrm{CP}\ \mathrm{intake}\hbox{-} \mathrm{IAAF}\  \mathrm{or}\  \mathrm{ICPF}\right)/\mathrm{Diet}\ \mathrm{AA}\ \mathrm{or}\ \mathrm{CP}\ \mathrm{intake}. $$

$$ \mathrm{SID}=\left(\mathrm{diet}\ \mathrm{AA}\ \mathrm{or}\ \mathrm{CP}\ \mathrm{intake}\hbox{-} \left[\mathrm{IAAF}\  \mathrm{or}\  \mathrm{ICPF}\hbox{-} \mathrm{EIAAF}\  \mathrm{or}\  \mathrm{ECPF}\right]\right)/\mathrm{Diet}\ \mathrm{AA},\mathrm{or}\ \mathrm{CP}\ \mathrm{intake} $$

where, EIAAF is the endogenous ileal amino acid flow, and ECPF is the endogenous crude protein flow calculated using Eq. 1 from the ileal digesta of chicks fed NFD.

### Statistical analysis

One-way ANOVA and general linear model (GLM) of Minitab software version 17 [39], were used to analyse the data. The effects of the main factors (RFFS level and enzyme supplementation as well as their interactions) were assessed. Differences between the mean values were separated by the Duncan’s multiple range tests and were considered to be significant at *P* ≤ 0.05.

## Results

### Response to the diets

The nutrient composition and quality measures for RFFS and SBM were different (Table 3). For examples, both the ether extracts (147.3 g/kg) and ME/kg (12.6 MJ ME/kg) contents of RFFS were higher than those of SBM, which were 19.2 g/kg and 9.0 MJ ME/kg; the reverse was the case for AA profile while the concentrations of TI and UA were lower in SBM than in the RFFS. Replacing the commercial SBM by RFFS from 0 to 75 g/kg diet resulted in an increase in selected ANF in the diets (Table 4). The TI concentration was increased from 1747.0 to 10,193.4 TIU/g; the NSI increased from 155.3 to 222.9 g/kg, and the UA was raised from 0.16 to 1.53 ∆pH.

**Table 3:** Analysed nutrient composition and quality parameters of raw soybean meal (RFFS) in comparison to the commercial soybean meal (SBM)

Item	Nutrient composition (g/kg)								Quality parameters (g/kg)								
Dry matter		Crude fibre	Crude protein	Ether extracts	Total Sugars	Starch	ME, MJ/kg		Available lysine		KOH	Trypsin inhibitors, TIU/g		Urease activity ΔpH			
RFFS	923.6	62.0	382.4	147.3	95.0	26.1	12.6		26.4		898.6	13,498		2.1			
SBM	914.8	37.9	422.9	19.2	107.6	37.0	9.0		28.4		794.4	5743		0.09			
	Indispensable amino acids										Dispensable amino acids						
	Met	Lys	Iso	Leu	Cys	Thr	His	Try	Arg	Val	Ala	Ser	Gly	Try	ASP	Glu	Pro
RFFS	5.6	26.6	17.9	31.0	6.0	16.1	10.9	4.7	32.9	18.7	17.3	19.4	17.3	4.7	45.8	73.3	20.2
SBM	6.2	29.0	20.5	35.6	6.0	18.2	12.1	6.5	32.9	21.9	20.3	20.2	19.5	6.5	51.0	82.5	22.7

*ME* metabolizable energy, *KOH* protein solubility. *RFFS* sample of raw full-fat soybean. *SBM* commercial soybean meal

**Table 4:** Effects of partially replacing commercial SBM by RFFS on quality of the diets

	RFFS (g/kg of diet)		
	0	45	75
Available lysine, g/kg	16.2	15.2	15.45
Nitrogen solubility, g/kg	155.3	187.6	222.9
Trypsin inhibitor (TIU/g)	1747	7897	10,194
Urease activity (∆pH)	0.16	1.00	1.525

The performance of the birds, in terms of FI, BWG and FCR are presented in Table 5. There were no significant (*P* > 0.05) interaction effects between RFFS and protease on the FI, BWG or FCR of birds during any of the assessed periods.

**Table 5:** Effects of protease in diets with raw soybean on FI, BWG (g/b) and FCR, in periods of day 0 to10, day 0 to 24 or day 0 to 35

RFFS^1^ g/kg	Protease PROT/kg	Feed intake			Body weight gain			FCR		
		0–10	0–24	0–35	0–10	0–24	0–35	0–10	0–24	0–35
0	0	315.7	1774	3608	290.4	1364	2626	1.09	1.28	1.36
	15,000	317.1	1804	3552	296.5	1470	2631	1.07	1.23	1.35
45	0	294.2	1724	3425	279.4	1335	2566	1.05	1.29	1.39
	15,000	306.1	1663	3322	274.9	1402	2537	1.12	1.22	1.34
75	0	294.3	1735	3426	266.3	1330	2444	1.12	1.31	1.43
	15,000	291.2	1708	3481	264.6	1376	2516	1.10	1.24	1.35
SEM		4.3	22.2	37.5	3.8	16.7	28.5	0.02	0.01	0.02
Main effects										
0		316.4	1789	3580^a^	293.5^a^	1417^a^	2629^a^	1.079	1.26	1.35
45		300.1	1693	3456^b^	277.2^ab^	1369^b^	2550^ab^	1.084	1.24	1.36
75		292.7	1707	3378^b^	265.5^b^	1338^b^	2473^b^	1.109	1.27	1.39
	0	301.4	1744	3490	278.7	1343^b^	2539	1.087	1.28^a^	1.39
	15,000	304.8	1716	3459	278.7	1406^a^	2563	1.095	1.23^b^	1.35
Sources of variation										
RFFS		0.08	NS	*	**	*	*	NS	NS	NS
Protease		NS	NS	NS	NS	*	NS	NS	*	NS
RFFS x protease		NS	NS	NS	NS	NS	NS	NS	NS	NS

^a,b^Means bearing uncommon superscripts within a column are significantly different at NS = non-significant; *P < 0.05; **P < 0.01; FI = feed intake; BWG = body weight gain; FCR = feed conversion ratio; ^1^RFFS = raw full-fat soybean (SBM was replaced by RFFS at zero, 15 and 25%, equivalent to zero, 45 and 75 g/kg of diet, respectively); SEM = standard error of means. A 15000 PROT/kg = 15,000 units of protease/kg of diet, equivalent to 200 mg of protease/kg of diet

The feed intake of birds was reduced with increase in RFFS inclusion in the diets, particularly affecting birds over the longer rearing period (day 0 to 35) (*P* < 0.05). Application of protease in these diets appeared not to influence (*P* > 0.05) the feed consumption. The FI was generally reduced (*P* < 0.05) during day 0 to 35 because of increasing inclusion of dietary RFFS in diets. The BWG was also decreased during day 0 to10 (*P* < 0.01), day 0 to 24 (*P* < 0.05) and day 0 to 35 (*P* < 0.05).

However, protease supplementation improved (*P* < 0.05) both the BWG and the FCR of birds during the day 0 to 24. Increasing the level of RFFS in diets (without protease supplementation) reduced the feed efficiency by 2.94%, whereas due to supplementation with microbial protease numerically improved the feed efficiency by 3.30%. However, these slight improvements were not statistically significant (*P* > 0.05). There were no significant (*P* > 0.05) effects of treatment on mortality.

#### Visceral organ weights

As shown in Table 6, the RFFS by protease interaction had no significant (*P* > 0.05) effects on the weight of any of the internal organs assessed at d 24. Increasing the RFFS inclusion rate in diets significantly increased the weight of the gizzard and proventriculus (*P* < 0.001), pancreas (*P* < 0.001), small intestine (jejunum + ileum + duodenum) (*P* < 0.001), heart (*P* < 0.001) and spleen (*P* < 0.05). The weight of the bursa also tended (*P* = 0.09) to increase at day 24. Protease supplementation significantly increased (*P* = 0.05) bursa weight but had no significant (*P* > 0.05) effects on any of the other measured internal organs.

**Table 6:** Effects of supplemental protease in diets containing graded levels of raw soybean on the weights of internal organs (g/ 100 g body weight) at day 24

RFFS^1^, g/kg	Protease, PROT/kg	G + P	Pancreas	SI	Heart	Liver	Bursa	Spleen
0	0	2.74	0.21	4.04	0.58	2.49	0.19	0.08
	15,000	2.79	0.21	4.02	0.54	2.66	0.20	0.08
45	0	3.72	0.36	5.43	0.69	3.07	0.20	0.12
	15,000	3.58	0.35	5.77	0.74	2.76	0.28	0.10
75	0	3.40	0.37	4.97	0.66	2.58	0.18	0.09
	15,000	3.31	0.35	4.71	0.64	2.87	0.20	0.08
SEM		0.10	0.02	0.19	0.02	0.09	0.01	0.01
Main effects								
0		2.77^c^	0.21^b^	4.03^b^	0.56^c^	2.58	0.20	0.08^b^
45		3.65^a^	0.35^a^	5.57^a^	0.71^a^	2.91	0.24	0.11^a^
75		3.36^b^	0.36^a^	4.84^a^	0.65^b^	2.73	0.19	0.08^b^
	0	3.29	0.31	4.81	0.64	2.7	0.19^b^	0.09
	15,000	3.23	0.30	4.71	0.64	2.8	0.23^a^	0.09
Sources of variation								
RFFS		***	***	***	***	NS	NS	*
Protease		NS	NS	NS	NS	NS	*	NS
RFFS x protease		NS	NS	NS	NS	NS	NS	NS

^a,b,c^Means bearing uncommon superscripts within a column are significantly different at NS = non-significant; ***P < 0.001; *P < 0.05; ^1^RFFS = raw full-fat soybean (SBM was replaced by RFFS at zero, 15 and 25%, equivalent to zero, 45 and 75 g/kg of diet, respectively); SEM = standard error of means; SI = Small intestine (jejunum, ileum and duodenum) were weighed with the contents; G + P (gizzard and proventriculus) were weighed with the contents; SEM = Pooled standard error of mean. A 15000 PROT/kg = 15,000 units of protease/kg of diet, equivalent to 200 mg of protease/kg of diet

#### Ileal digestibility of crude protein and amino acids.

The results revealed that basal endogenous loss of ileal CP was significantly (*P* < 0.001) increased in response to rising the level of RFFS. On average, the basal endogenous loss of ileal AA, except that of methionine at day 24 was significantly increased in birds fed diets containing RFFS (Table 7).

**Table 7:** Effects of protease supplementation of diets containing raw soybean on the ileal flow (g/kg of FI) of undigested crude protein and amino acids (mg/g) at day 24

RFFS^1^(g/kg)	Protease (PROT/kg)	CP	Indispensable amino acids										Dispensable amino acids			
			His	Arg	Thr	Lys	Met	Val	Ile	Leu	Phe		Ser	Gly	Ala	Pro
0	0	41.30	0.96	1.47	2.06	1.97	0.28	2.32	1.86	3.30	1.89		2.26	2.94	2.21	2.82
	15,000	38.50	0.87	1.40	1.95	1.75	0.24	2.05	1.65	2.88	1.67		2.11	2.82	1.96	2.63
45	0	43.60	1.01	1.56	2.22	2.09	0.26	2.47	2.02	3.52	2.03		2.49	3.03	2.34	2.99
	15,000	40.60	1.02	1.56	2.23	2.03	0.30	2.46	1.99	3.50	1.97		2.49	3.02	2.29	2.93
75	0	65.30	1.12	1.82	2.36	2.33	0.31	2.78	2.30	3.99	2.28		2.71	3.26	2.53	3.11
	15,000	55.60	1.09	1.70	2.35	2.15	0.30	2.68	2.20	3.90	2.16		2.65	3.24	2.51	3.16
SEM		0.9	0.02	0.05	0.04	0.05	0.01	0.06	0.05	0.10	0.05		0.05	0.04	0.06	0.06
Main effects																
0		39.90^b^	0.92^b^	1.43^b^	2.01^b^	1.86^b^	0.26	2.19^c^	1.75^c^	3.09^c^	1.78^c^		2.18^b^	2.88^b^	2.09^b^	2.72^b^
45		42.10^b^	1.01^b^	1.56^a^	2.22^a^	2.06^a^	0.28	2.46^b^	2.01^b^	3.51^b^	2.00^b^		2.49^a^	3.03^b^	2.32^ab^	2.96^ab^
45		60.50^a^	1.10^a^	1.76^a^	2.35^a^	2.24^a^	0.30	2.73^a^	2.25^a^	3.94^a^	2.22^a^		2.68^a^	3.25^a^	2.52^a^	3.14^a^
	0	49.10	1.03	1.61	2.22	2.11	0.29	2.52	2.05	3.60	2.05		2.48	3.07	2.34	2.95
	15,000	45.90	0.99	1.55	2.17	2.00	0.27	2.40	1.96	3.43	1.95		2.42	3.03	2.27	2.93
Sources of variation																
RFFS		***	**	*	**	**	NS	***	***	***	***		***	***	**	**
Protease		NS	NS	NS	NS	NS	NS	NS	NS	NS	NS		NS	NS	NS	NS
RFFS x protease		NS	NS	NS	NS	NS	NS	NS	NS	NS	NS		NS	NS	NS	NS

^a,b,c^Means bearing uncommon superscript within a column are significantly different at NS = non-significant; *P < 0.05; **P < 0.01; ***P < 0.001; ^1^RFFS = raw full-fat soybean (SBM was replaced by RFFS at zero, 15 and 25%, equivalent to zero, 45 and 75 g/kg of diet, respectively); SEM = standard error of means. A 15000 PROT/kg = 15,000 units of protease/kg of diet, equivalent to 200 mg of protease / kg of diet

At day 24 d, increasing the RFFS inclusion rate significantly reduced (*P* < 0.01) the values of AID and SID for CP, and it also reduced the value of AID and SID of indispensable AA by up to 8.5 and 7.7%, respectively, with the lowest value for methionine and the highest for isoleucine. The AID and SID values of dispensable AA were also reduced by between 5.0 to 8.0 and 4.0 to 7.0%, respectively in line with increase in RFFS (Tables 8 and 9).

**Table 8:** Effects of protease and RFFS supplementations on the coefficient of apparent ileal digestibility of CP and AA of broilers at day 24

RFFS^1^ (g/kg)	Protease (PROT/kg)	CP	Indispensable amino acids									Dispensable amino acids			
			His	Arg	Thr	Lys	Met	Val	Ile	Leu	Phe	Ser	Gly	Ala	Pro
0	00	0.775^b^	0.818	0.905	0.762	0.848^a^	0.945	0.786	0.800	0.824	0.825	0.793	0.770	0.815	0.806
	15,000	0.812^a^	0.841	0.894	0.772	0.859^a^	0.948	0.802	0.809	0.832	0.832	0.794	0.748	0.815	0.801
45	0	0.783^ab^	0.815	0.888	0.755	0.838^ab^	0.946	0.762	0.771	0.801	0.807	0.761	0.712	0.779	0.777
	15,000	0.772^b^	0.816	0.891	0.754	0.848^a^	0.952	0.774	0.782	0.810	0.808	0.771	0.737	0.792	0.784
75	0	0.757^b^	0.785	0.867	0.721	0.810^b^	0.941	0.727	0.724	0.780	0.773	0.737	0.692	0.768	0.766
	15,000	0.776^b^	0.799	0.880	0.744	0.837^ab^	0.943	0.743	0.750	0.784	0.789	0.745	0.706	0.771	0.765
SEM		0.004	0..04	0.004	0..04	0.004	0..02	0.006	0.007	0.006	0.005	0.005	0.006	0.005	0.005
Main effects															
0		0.794	0.830^a^	0.899^a^	0.767^a^	0.854	0.946	0.794^a^	0.805^a^	0.828^a^	0.828^a^	0.793^a^	0.759^a^	0.815^a^	0.804^a^
45		0.778	0.816^b^	0.889^ab^	0.755^ab^	0.843	0.949	0.768^b^	0.777^b^	0.805^ab^	0.807^ab^	0.766^b^	0.725^b^	0.785^b^	0.781^b^
75		0.766	0.792^b^	0.874b	0.733^b^	0.823	0.942	0.735^c^	0.737^c^	0.782^b^	0.781^b^	0.741^c^	0.699^c^	0.769^c^	0.765^c^
	0	0.772	0.806	0.887	0.746	0.832	0.944	0.758	0.765	0.802	0.802	0.764	0.725	0.787	0.783
	15,000	0.786	0.819	0.888	0.757	0.848	0.948	0.773	0.781	0.808	0.809	0.770	0.730	0.793	0.783
Sources of variation															
RFFS		**	***	**	***	**	NS	***	***	***	***	***	***	***	***
Protease		*	0.08	NS	NS	*	NS	NS	NS	NS	NS	NS	NS	NS	NS
RFFS x protease		*	NS	NS	NS	*	NS	NS	NS	NS	NS	NS	NS	NS	NS

^a,b,c^ Means bearing uncommon superscripts within a column are significantly different at NS = non-significant; *P < 0.05; **P < 0.01; ***P < 0.00; ^1^RFFS = raw, full-fat soybean (SBM was replaced by RFFS at zero, 15 and 25%, equivalent to zero, 45 and 75 g/kg of diet, respectively); SEM = standard error of means. A 15000 PROT/kg = 15,000 units of protease/kg of diet, equivalent to 200 mg of protease / kg of diet

**Table 9:** Effects of protease and RFFS supplementations on the coefficient of standardized ileal digestibility of CP and AA of broilers at day 24

RFFS^1^ (g/kg)	Protease (PROT/kg)	CP	Indispensable amino acids									Dispensable amino acids			
			His	Arg	Thr	Lys	Met	Val	Ile	Leu	Phe	Ser	Gly	Al	Pro
0	0	0.804^b^	0.873	0.932	0.851	0.887	0.966	0.847	0.853	0.864	0.867	0.860	0.828	0.870	0.853
	15,000	0.840^a^	0.894	0.921	0.858	0.896	0.968	0.860	0.861	0.871	0.872	0.859	0.811	0.872	0.848
45	0	0.811^ab^	0.868	0.916	0.837	0.875	0.963	0.821	0.824	0.840	0.848	0.827	0.780	0.837	0.826
	15,000	0.799^b^	0.869	0.918	0.839	0.884	0.971	0.832	0.833	0.848	0.848	0.836	0.800	0.848	0.831
75	0	0.785^b^	0.841	0.897	0.810	0.850	0.961	0.789	0.780	0.819	0.815	0.806	0.761	0.826	0.815
	15,000	0.803^b^	0.853	0.907	0.825	0.873	0.962	0.803	0.802	0.823	0.830	0.812	0.770	0.828	0.813
SEM		0.004	0.005	0.004	0.004	0.004	0.002	0.006	0.007	0.006	0.005	0.005	0.006	0.004	0.005
Main effects															
0		0.822	0.884^a^	0.927^a^	0.855^a^	0.891^a^	0.967	0.853^a^	0.857^a^	0.868^a^	0.869^a^	0.859^a^	0.819^a^	0.871^a^	0.851^a^
45		0.805	0.869^a^	0.917^b^	0.838^b^	0.879^b^	0.967	0.827^b^	0.829^b^	0.844^b^	0.848^b^	0.831^b^	0.790^b^	0.843^b^	0.829^b^
75		0.794	0.847^b^	0.902^c^	0.818^b^	0.861^c^	0.961	0.796^c^	0.791^c^	0.821^b^	0.822^c^	0.809^c^	0.765^c^	0.827^b^	0.814^b^
	0	0.800	0.861	0.915	0.833	0.871^b^	0.963	0.819	0.819	0.841	0.843	0.831	0.789	0.844	0.831
	15,000	0.814	0.872	0.915	0.841	0.884^a^	0.967	0.831	0.832	0.847	0.850	0.836	0.793	0.849	0.831
Sources of variation															
RFFS		**	***	***	***	**	NS	***	***	**	***	***	***	***	***
Protease		*	NS	NS	NS	*	NS	NS	NS	NS	NS	NS	NS	NS	NS
RFFS x protease		*	NS	NS	NS	NS	NS	NS	NS	NS	NS	NS	NS	NS	NS

^a,b,c^Means bearing uncommon superscripts within a column are significantly different at NS = non-significant; *P < 0.05; **P < 0.01; ***P < 0.001; ^1^RFFS = raw full-fat soybean (SBM was replaced by RFFS at zero, 15 and 25%, equivalent to zero, 45 and 75 g/kg of diet, respectively); SEM = standard error of means. A 15000 PROT/kg of diet = 15,000 units of protease/kg of diet, equivalent to 200 mg of protease/kg of diet

Under microbial protease supplementation, the basal endogenous loss of ileal CP and total AA were reduced by approximately 7.0 and 3.5%, respectively, but the differences were not significant (*P* > 0.05). The AID and SID of CP measured at day 24 were significantly (*P* < 0.05) increased when the diets were supplemented with microbial protease, and they were also significantly (*P* < 0.05) influenced by the interaction effects between protease and RFFS. Protease supplementation had the reverse effect on the AID and SID of CP, resulting into a lack of interaction between the two main factors.

Although statistically the same (*P* > 0.05), the average basal endogenous loss of indispensable and dispensable AA at the ileum, were reduced by approximately 4.5 and 2.0%, respectively when the diets were supplemented with protease. However, supplementation with protease resulted in an increase in the AID and SID values of indispensable AA, which respectively ranged between 0 and 2.0% and 0 and 1.5% more than the non-supplemented diets, but the differences were not statistically significant (*P* > 0.05). Although the differences were not significant (*P* > 0.05), the average AID and SID values of dispensable AA at day 24 were 0.78% and 0.56%, respectively greater when the diets were supplemented with microbial protease. The AID (*P* < 0.5) and SID (*P* < 0.05) values of lysine were significantly increased due to supplementation of diets with that of microbial protease.

## Discussion

### Diets, performance parameters and internal organ development

The variations in nutrient contents between the samples of RFFS used in this and other studies may be due to various reasons, including crop variety, processing, and geographical origin [40, 41]. The lower contents of AA in the RFFS in the current study may be largely due to the high fat (oil) content of the material.

The response of birds in terms of FI, BWG and FCR was affected over the 0 to 35-day period due to the high concentration of TI in the dietary treatments, with one diet close to 10,200 TIU/g, which is beyond the threshold level for non-ruminant animals [42]. However, significant improvements were observed in BWG and FCR during day 0 to 24 due to microbial protease supplementation. The current results are consistent with those of other researchers [1, 43] who reported that protease can break down both the stored proteins and the protein-like anti-nutrients and subsequently improve nutrient digestibility. Although not investigated in the current study, the exogenous protease may have complemented the effects of endogenous enzymes, and altered the digestibility of nutrients and possibly feed passage rate [44, 45].

The weight of most of the internal organs of the birds, including the gizzard and proventriculus, pancreas, small intestine (SI), heart and spleen, was increased by increasing inclusion level of RFFS in diets. This finding partially agrees with those of other researchers [46, 47] who reported that birds fed diets containing RFFS had heavier pancreas and duodenum. The reasons for the increased weights of these internal organs may be a response in form cellular hypertrophy or hyperplasia. The crude fibre content of RFFS was higher than that of commercial SBM and be intact due to lack of processing. In a previous study, we observed an increased weight of the pancreas in birds fed diets containing RFFS [48]. The small intestine, particularly duodenum is anatomically close to the pancreas and it was also similarly affected because of increasing levels of RFFS in diets. This result is supported by reports of other researchers [49]. The relationship between the body weight and visceral organs, in general needs to be considered as the former was reduced by the effect of RFFS, so that the relative weight of the latter became accentuated.

### Ileal digestibility of amino acids and crude protein

Increasing the level of RFFS in diets significantly increased the loss of basal endogenous loss of ileal CP and AA; consequently, reducing the AID and the corresponding SID. These results are inconsistent with those of Clarke and Wiseman [12] who reported that the AID and SID of AA did not correlate with TI levels. The digesta collected from the ileum may contain both dietary undigested materials and endogenous protein and AA [50]. However, the results agree with that of de-Coca-Sinova et al. [51] who reported that the apparent digestibility of N and AA in broilers varies with SBM samples, with greater values corresponding to lower concentration of TI in diets. Moreover, Barth et al. [52] explained that the ingestion of food containing TI influenced N balance by increasing the outflow of amino acids from endogenous secretions rather than through the loss of dietary amino acids.

The AID and SID of most dispensable and indispensable amino acids assessed at d 24 were significantly reduced by increasing inclusion level of RFFS. These results are in contrast with those of Frikha et al. [53] who reported that the SID of CP and lysine in broilers was increased at day 21 due to the inclusion of soybeans with high KOH and TIA values. The current results are supported by other researchers [54] who reported a reduction on the apparent digestibility of nutrients when raw soybean meal was fed to broiler chicks. Similarly, Gilani et al. [8] indicated that the high concentrations of ANF in diets from grain legumes are responsible for poor digestibility of protein. The reduction in AID and SID of CP and AA in the current study may be linked to the increased loss of basal endogenous ileal CP and AA. However, when the diets were supplemented with microbial protease, the basal endogenous loss of ileal CP was reduced. This led to increase in the AID and SID of CP although this was not significant. The current finding partially agree with those of previous researchers [55–57] who observed an increase in AID of AA in poultry and piglets and health benefits in response to inclusion of microbial protease in the diets.

Protease supplementation significantly improved the AID and SID of lysine. This partially agrees with the finding of Liu et al. [58] who observed a 9% improvement in apparent digestibility of AA in broilers fed a corn-sorghum-based diet when supplemented with protease. It is not clear why lysine was the only indispensable AA to significantly respond in the current study but it may be related to its digestibility status in RFFS. No reasons could be proffered for the lack of effect of the test product on the digestibility of methionine, which was not affected.

## Conclusion

This study showed that some commercial SBM could be replaced (≤25%) by RFFS in broiler diets if the diets are supplemented with the right protease. Body weight gain seemed to be the most affected by the high levels of TI. It is evident from the present study that the test microbial protease could reduce the adverse impact of dietary ANF, particularly TI, on the body weight gain and feed efficiency during up to the end of the grower phase. One major area of action of the protease appears to be the reduction in the basal endogenous loss of CP and AA at the ileum, leading to an increase in the AID and SID of CP and AA. Further studies may be required to establish the direct impact of the test protease on RFFS protein and the differing responses that were observed for methionine and lysine.

## Abbreviations

AA: Amino acids
AEC: Animal Ethics Committee
AID: Apparent ileal digestibility
ANF: Anti-nutritional factors
BWG: Body weight gain
CP: Crude protein
d: day
ECPF: Endogenous crude protein flow
EIAAF: Endogenous ileal amino acid flow
FCR: Feed conversion ratio
FI: Feed intake
GLM: General linear model
IAAF: Ileal amino acids outflow
ICPF: Ileal crude protein outflow
ME: Metabolizable energy
MJ: Mega joule
N: Nitrogen
NFD: Nitrogen-free diet
NSI: Nitrogen solubility index
RFFS: Raw full fat soybean
SBM: Soybean meal
SI: Small intestine
SID: Standardized ileal digestibility
Ti: Titanium
TI: Trypsin inhibitors
TIU: Trypsin inhibitors unit
UA: urease activity
